# App-supported versus conventional college physical education: Effects on standardized physical fitness scores and exercise motivation in Chinese university students

**DOI:** 10.1371/journal.pone.0345759

**Published:** 2026-03-23

**Authors:** Xilin Liang, Zenan Wang

**Affiliations:** 1 School of Humanities, Zhejiang Business College, Hangzhou, Zhejiang, China; 2 Department of Physical Education, Dankook University, Yongin-si, Republic of Korea; University of Tartu, ESTONIA

## Abstract

This study evaluated the effectiveness of an app-supported training model versus conventional physical education (PE) on college students’ basic athletic abilities and described exercise motivation in the app-supported condition. A quasi-experimental study was conducted with 90 freshman students over a 10-week period. Participants were assigned to a Treatment Group (n = 45), using the “Campus Sports World” app for guided extracurricular training, and a Control Group (n = 45) following traditional instruction. Physical fitness was assessed pre- and post-intervention using standardized tests for speed, endurance, explosive power, and flexibility. Motivation was assessed post-intervention in the Treatment Group via a questionnaire based on Self-Determination Theory. Results indicated that the Treatment Group achieved significantly greater improvements in speed, explosive power, and flexibility compared to the Control Group. However, no significant difference was found in endurance performance. Furthermore, students in the Treatment Group reported high levels of both intrinsic and extrinsic motivation. These findings suggest that integrating mobile applications into college PE can enhance skill-related fitness, and the observed motivation profile provides context for students’ engagement with the app-supported model, supporting the adoption of blended learning approaches in higher education.

## Introduction

College students’ physical fitness has become a major public health and educational concern. Recent large-scale investigations have documented a gradual decline in the physical fitness levels of Chinese university students, with noticeable deficits in speed, endurance, explosive power and flexibility compared with earlier cohorts [1]. In response, the Chinese government has elevated physical fitness to a national strategic priority and issued successive national fitness plans, including the National Fitness Program (2016–2020), to promote regular physical activity and improve population health [2]. Within the education system, the National Student Physical Health Standard (2014 revision) specifies standardized fitness tests for university students—such as the 50 m sprint, 800/1000 m run, standing long jump and sit-and-reach—as compulsory indicators used to evaluate students’ physical health and, in many institutions, to inform academic decisions [3].

In parallel with these policy developments, mobile and digital technologies have been rapidly integrated into college physical education (PE). Universities increasingly rely on app-based platforms to record students’ exercise behaviors, monitor training load and manage fitness test data. Campus Sports World is one of the most widely adopted systems of this kind in Chinese higher education, and many institutions now use its records as an important component of end-of-term PE grades. Studies on technology-enhanced physical education have shown that digital assessment platforms, wearable devices and fitness applications can support data collection, feedback and performance evaluation, and may improve students’ engagement and fitness outcomes [4–6]. For example, Cheng and Yin developed a computer-based physical education examination platform that streamlines data processing and scoring [5], while Anthony synthesized international evidence showing that integrating technologies such as fitness trackers, interactive applications and virtual tools into PE can enhance student participation and fitness [4]. Other work has explored more advanced solutions, such as augmented-reality and IoT-enabled systems, which provide real-time feedback and immersive training environments in school physical education [6].

Despite this growing body of research, several gaps remain. First, empirical evidence on the effectiveness of specific, widely used platforms in real university settings is still limited. In particular, few studies have rigorously compared app-supported training models with conventional college PE instruction in terms of objectively measured changes in basic athletic abilities such as speed, endurance, explosive power and flexibility. Second, prior work has focused primarily on physical performance outcomes, with far less attention to how app-based systems affect students’ motivation for sports learning—especially the interplay between intrinsic motivation and extrinsic motivation in a high-stakes evaluation context.

Against this background, the present study takes Campus Sports World as a representative example of an app-based system embedded in Chinese college PE and examines both physical fitness and motivational outcomes. Drawing on the task–technology fit perspective and research on exercise motivation, the study compares an app-supported training model with conventional PE instruction that does not rely on the platform. By combining standardized fitness tests with validated measures of intrinsic and extrinsic motivation, it aims to provide data-based evidence regarding the educational value of using Campus Sports World data in formal assessment. More specifically, this study addresses the following research questions:

**RQ1:** Compared with conventional college PE instruction, does participation in a Campus Sports World–supported training model lead to greater improvements in students’ basic athletic abilities-specifically speed, endurance, explosive power and flexibility-as measured by standardized physical fitness tests?

**RQ2:** What is the profile of intrinsic and extrinsic motivation among students participating in the Campus Sports World–supported training model?

### Theoretical framework

#### Task–technology fit perspective.

Task–technology fit (TTF) provides the first theoretical lens for this study. TTF holds that the impact of an information system on performance depends on the extent to which its functional capabilities match the requirements of the tasks it is intended to support [7]. When technology characteristics (e.g., data recording, feedback, usability) are well aligned with task characteristics (e.g., complexity, information needs, performance standards), users can complete their work more efficiently and effectively. Conversely, even sophisticated technologies may fail to improve outcomes if there is a poor fit between what the system provides and what the task demands.

Subsequent research has extended TTF from traditional office information systems to learning management systems and other educational technologies, showing that fit between technology features and pedagogical tasks is a key determinant of perceived impact and learning outcomes [8]. In these contexts, task–technology fit is typically reflected in whether the system supports core teaching and learning activities—such as content delivery, progress monitoring, feedback and assessment—in ways that correspond to course objectives and institutional requirements.

In this study, TTF offers a performance-oriented rationale for comparing an app-supported training model with conventional PE instruction that does not use Campus Sports World. We propose that the app enhances the fit between the demands of extracurricular PE training and the technological support available to students. Specifically, the app can support key training requirements, including scheduling, monitoring, feedback, and documentation. When task–technology fit is improved, students may be better positioned to plan, organize, and carry out training tasks, which in turn may be reflected in measurable gains in physical fitness during the intervention period. Since TTF primarily addresses functional alignment and task execution, we draw on SDT as a complementary framework to describe and interpret the motivational profile observed in the app-supported learning environment.

#### Exercise motivation in college physical education.

The second theoretical lens concerns exercise motivation. Self-determination theory (SDT) distinguishes between intrinsic motivation—engaging in an activity for its inherent interest, enjoyment or challenge—and extrinsic motivation—engaging in an activity because it leads to separable outcomes such as grades, rewards or social approval [9,10]. Within SDT, the quality of motivation is closely linked to the satisfaction of three basic psychological needs: autonomy, competence and relatedness. When these needs are met, individuals are more likely to develop autonomous forms of motivation and to persist in health-related behaviors such as regular exercise.

Applied to physical education and exercise behavior, SDT research has repeatedly linked more self-determined motivation to greater exercise adherence, sustained effort, and longer-term participation [11,12]. Among university students, autonomous motivation is typically associated with higher levels of physical activity engagement and, in turn, more favorable fitness outcomes [13,14]. To further explain how motivation fostered in educational settings may extend to out-of-class exercise, the trans-contextual model proposes that autonomous motivation developed in PE can transfer across contexts and support extracurricular physical activity engagement [15]. In the present study, SDT is used to frame and interpret students’ intrinsic and extrinsic motivation under the app-supported condition. Motivation, however, was assessed only once after the intervention and only in the Treatment Group. Therefore, the study does not examine SDT-based causal pathways that would connect need support or need satisfaction to motivation, behavior, and subsequent fitness changes. For this reason, motivation results are reported as exploratory and descriptive evidence that helps contextualize the intervention experience, rather than as explanatory mechanisms.

In addition to self-determination theory, the trans-contextual model of autonomous motivation, abbreviated as TCM, is relevant to the present study because it explains how motivation cultivated in physical education can transfer to students’ leisure-time physical activity. A meta-analysis summarizes evidence that autonomy-supportive teaching in education settings is linked with more self-determined motivation and stronger intentions and behavior beyond the classroom [15]. In college samples, empirical work has also shown that autonomy support in physical education is associated with autonomous motivation toward leisure-time physical activity, and intervention work has used TCM-based course design to promote leisure-time activity behavior among university students. These findings provide a useful theoretical bridge for discussing extracurricular training engagement in app-supported PE contexts, even though the current study does not test motivational transfer pathways directly.

#### Conceptual model and hypotheses.

Building on TTF and SDT, we use an integrated conceptual model. In this model, Campus Sports World is expected to influence physical fitness mainly by enhancing the task–technology fit of extracurricular training, while SDT offers a complementary perspective for describing and interpreting students’ motivational orientations in an app-supported setting.

From a TTF perspective, Campus Sports World is treated as an information system whose key functions-standardized tutorials, training monitoring and recording, goal setting, and feedback-map onto core requirements of college PE training. When the fit is higher, students may face fewer execution barriers and manage their training more effectively. Because the app foregrounds structured practice, technique demonstrations, and timely performance feedback, we expect stronger effects on skill-related components such as speed, explosive power, and flexibility than on endurance, which typically depends on sustained overload and more precise control of training intensity and volume.

From an SDT perspective, the app-supported environment may be experienced as more need-supportive, promoting autonomy, competence, and relatedness. This may occur through progress visualization, self-paced task completion, and opportunities for peer interaction and comparison. Consistent with the study design, we do not use SDT to test mediational mechanisms. Instead, we report post-intervention intrinsic and extrinsic motivation among participants in the Treatment Group to help contextualize their intervention experience. Based on this framework, the study formulates the following hypotheses:

**H1:** Students who participate in a Campus Sports World–supported training model (Treatment Group) will show greater improvements in standardized physical fitness test scores-specifically speed, endurance, explosive power, and flexibility-than students who receive conventional college PE instruction without the app (Control Group).

**H2:** Within the Treatment Group, mean levels of intrinsic and extrinsic motivation will be above the midpoint of the scale, reflecting students’ motivation profile in the app-supported condition.

## Materials and methods

### Participants

A total of 90 first-year undergraduates from the Art and Design program at Zhejiang Business College were recruited for this study. We employed purposive sampling to select two intact parallel classes with comparable gender distributions. Eligible students were those who had completed standard high school physical education and met the university’s general health requirements for compulsory PE courses. Notably, none of the participants had a background in professional sports training or any pre-existing medical conditions that might limit their physical activity. The recruitment phase and subsequent data collection spanned from September 15, 2025, to November 20, 2025.

### Ethical considerations

The research protocol followed the ethical principles of the Declaration of Helsinki and received formal approval from the Institutional Review Board (Ethics Committee) of Zhejiang Business College. Because the physical tests involved, including the 50 m sprint, standing long jump, 800 m/1000 m run, and sit-and-reach, are standard components of the National Student Physical Health Standard, the study was categorized as minimal risk. Consequently, the Ethics Committee permitted oral informed consent to maintain a natural teaching environment, minimize administrative interference, and avoid collecting additional written identifiers.

Prior to starting the experiment, we briefed all students on the study goals, the timeline, and the measures taken to ensure data anonymity. Participation was voluntary, and students were informed that they could withdraw at any stage without any impact on course grades or academic standing. Oral consent was obtained from each student, with the class sports representative acting as a witness and documenting the process in the researchers’ teaching logs. All personal identifiers were removed before analysis.

### Research design and procedures

**Fig 1:**
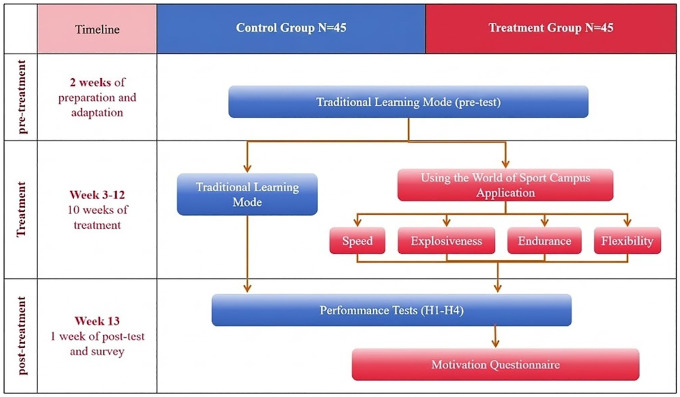
Study design and assessment timeline.

The overall experimental design and study procedures are summarized in Fig 1. We utilized a 10-week quasi-experimental pretest–posttest design. The two participating classes were assigned at the class level to either an experimental group (n = 45) or a control group (n = 45). To ensure consistency, the same instructor taught both classes, following the standard university syllabus with identical weekly hours and objectives. Individual randomization was not feasible due to teaching management requirements, because the university PE course was delivered in intact natural classes with pre-assigned rosters and schedules. The two classes represented different majors within the same year cohort, and interaction outside PE sessions was likely limited; nevertheless, minor spillover cannot be completely ruled out.

The intervention for the experimental group involved embedding the Campus Sports World app into the curriculum. The instructor used the app to assign weekly training tasks aligned with national health standards. Students recorded their extracurricular exercise sessions through the app’s interface, allowing the instructor to monitor progress and incorporate this data into the students’ final assessments. Meanwhile, the control group followed a traditional, teacher-led format. While the instructor encouraged these students to exercise outside of class, their activities were not tracked or evaluated through any digital platform.

Standardized physical fitness tests were administered to both groups one week before and one week after the intervention. Immediately following the post-test, students in the experimental group completed a brief paper-based questionnaire to assess their intrinsic and extrinsic motivation for sports learning within the app-supported context. The control group did not complete this survey as they had no exposure to the platform. To measure motivation, we used a validated exercise motivation scale specifically adapted for the college physical education environment. By comparing pre- and post-intervention changes in standardized physical fitness indicators between the two groups, we evaluated the impact of the Campus Sports World–supported model on students’ basic athletic abilities. Motivation data were used to describe the motivational profile in the app-supported condition and were not analyzed through between-group comparisons. Because the motivation questionnaire was designed for students after using Campus Sports World and all items explicitly refer to app use (Table 1), it was administered only once at posttest in the Treatment Group; no pre-intervention motivation assessment was conducted.

**Table 1:** Measurement items and sources for exercise motivation.

Construct	Measurement Items
Intrinsic Motivation	1. Using the app makes me more willing to exercise.
	2. I would use the app to learn more about sports if time permits.
	3. Learning new knowledge in the app brings me happiness.
	4. Through the app, I found new training methods/content.
	5. Knowledge from the app inspires my confidence to participate.
Extrinsic Motivation	6. When I make progress on the app, my enthusiasm increases.
	7. The app encourages me to complete tasks within the given time.
	8. My exercise motivation increases when using the app.
	9. Even if I dislike PE, I can get good grades by following the app.
	10. I want to get good grades to show my ability to others.

### Instruments

#### Campus sports world app.

Campus Sports World is a mobile application widely adopted in Chinese universities for managing students’ physical activity and physical fitness assessments. In the present study, it served as the core platform for organizing, recording and monitoring extracurricular exercise in the experimental group. Students installed the app on their smartphones and logged in with their institutional accounts.

According to the requirements of the National Student Physical Health Standard [3] and the course syllabus, the instructor released weekly extracurricular training tasks through the app. The tasks specified exercise modes, mainly running and aerobic activities, and set basic volume targets with a defined completion window. Weekly tasks were assigned at the class level and were the same for all students in the Treatment Group. The app was used for task release, recording, and completion checking rather than as an individualized training prescription system. During each session, students used the app’s GPS and step-counting functions to record their activity. The system automatically calculated duration and distance and stored cumulative training volumes. The app did not provide intensity prescriptions or objective intensity monitoring, such as heart-rate zones, pace targets, or wearable-based workload indicators, and these indicators were not collected in this study. The instructor checked students’ completion through the management interface, provided feedback, and used app-generated records as an important component of process evaluation in the Treatment Group. When students reported temporary discomfort, the instructor allowed volume reduction or rescheduling within the completion window on a case-by-case basis. The Control Group did not use Campus Sports World and had no app-based recording or monitoring of extracurricular exercise.

#### Physical fitness tests.

Students’ basic athletic abilities were assessed with the college version of the National Student Physical Health Standard (Ministry of Education of the People’s Republic of China, 2014), which is the official physical fitness evaluation system for Chinese university students. Four test items were selected to represent key components of physical fitness:(1) The 50 m sprint for speed;(2) The 800 m run for females and 1000 m run for males for aerobic endurance;(3) the standing long jump for lower-limb explosive power; and (4) the sit-and-reach test for trunk and hamstring flexibility.

All tests were administered by trained PE staff following the national standard procedures. Times were recorded to the nearest 0.1 s and distances to the nearest 0.1 cm, and converted into standardized scores based on sex- and age-specific norms. Pretests and posttests were conducted at the same venue and time of day, and the test order was kept consistent across groups and measurement occasions to minimize measurement error.

#### Exercise motivation questionnaire.

Exercise motivation in the context of college physical education was assessed with a questionnaire developed for the Campus Sports World environment. The instrument was grounded in self-determination theory (SDT), which distinguishes between intrinsic and extrinsic motivation [9,10]. Because the motivation items were app-referential and required app experience, they could not be meaningfully administered at baseline prior to app use.

To ensure content validity, items were adapted from validated scales in previous studies and modified to fit app-based physical education. As shown in Table 1, the scale consists of 10 items. Items 1–8 were mainly adapted from Asad et al. [16] to capture interest and task motivation, item 9 from Eom and Ashill [17] to reflect grade-oriented motivation, and item 10 from Tsai et al. [18] to capture motivation related to social recognition. All items were translated into Chinese and then back-translated by bilingual experts to ensure linguistic and conceptual equivalence. Because the items were phrased with explicit reference to app use, the questionnaire was administered only to the Treatment Group at posttest. Items were grouped a priori into two subscales to reflect SDT’s broad distinction between intrinsic and extrinsic motivation. Given that the instrument was assembled from multiple sources and tailored to an app-supported setting, we interpret the two subscales as indicators of broad motivational orientation rather than as fine-grained SDT regulations. Some statements related to progress and performance may also be consistent with identified or introjected reasons for participation, so the intrinsic–extrinsic distinction should be interpreted with this overlap in mind.

All items were rated on a five-point Likert scale ranging from 1 (“strongly disagree”) to 5 (“strongly agree”). Reliability analysis confirmed the internal consistency of the instrument. In the present study, both subscales demonstrated excellent reliability, with Cronbach’s alpha coefficients of 0.914 for intrinsic motivation and 0.883 for extrinsic motivation (see Table 2). Internal consistency was therefore satisfactory, while the construct validity of this brief, app-tailored measure warrants further verification in larger samples.

**Table 2:** Results of Cronbach’s alpha of the research instruments.

Variables	Number of Items	Cronbach’s Alpha
Intrinsic Motivation	5	0.914
Extrinsic Motivation	5	0.883

Preliminary construct validity of the exercise motivation questionnaire was examined using exploratory factor analysis. The results indicated acceptable sampling adequacy and a two-factor solution broadly consistent with intrinsic and extrinsic motivation. Given the relatively small sample size, the factor-analytic results are interpreted as exploratory. Detailed factor loadings and statistics are reported in the Supporting Information (S1 Table).

### Data collection procedure

Data for this study were collected within one teaching term at Zhejiang Business College. In the week before the intervention, the experimental class and the control class both completed the four standardized physical fitness tests (50 m sprint, standing long jump, 800 m run for females or 1000 m run for males, and sit-and-reach) during their regular PE period. The tests were organized by the researcher together with PE staff and were carried out on the outdoor track and field ground according to the National Student Physical Health Standard procedures.

During the following 10 weeks, the experimental class used the Campus Sports World app to complete and record their assigned extracurricular exercise tasks, while the control class received conventional PE instruction and was not required to use any app for exercise recording. In the final week of the term, the same set of physical fitness tests was administered again to both classes under similar conditions and at roughly the same time of day. Immediately after finishing the posttest, students in the experimental class were invited to complete a short paper-based questionnaire about their motivation and their experience with the app-supported training model. The control class did not complete this questionnaire because they had not used the app. All test scores and questionnaire responses were checked on site for completeness and then entered into an anonymized dataset for subsequent analysis. All 90 participants completed both pre- and posttests and there were no missing data.

### Data analysis

All quantitative analyses were conducted using IBM SPSS Statistics version 26.0. Raw scores from the four physical fitness tests (50 m sprint, 800/1000 m run, standing long jump, and sit-and-reach) were entered into SPSS and screened for data entry errors and missing values. Following the National Student Physical Health Standard (2014 revision), raw performances were converted into sex- and age-specific standardized scores so that outcomes could be compared on a common metric across students.

For the exercise motivation questionnaire, item responses were coded from 1 (strongly disagree) to 5 (strongly agree) and averaged to obtain subscale scores for intrinsic motivation and extrinsic motivation. Assumptions were evaluated using the Shapiro–Wilk test for normality and Levene’ s test for homogeneity of variance. Baseline equivalence and posttest between-group differences were examined using independent-samples t tests, and within-group pre–post changes were examined using paired-samples t tests. In addition, to account for the repeated-measures structure, we conducted 2 (Group: Treatment vs. Control) × 2 (Time: pretest vs. posttest) mixed-design ANOVAs for each fitness indicator, focusing on the Group×Time interaction effect.

Because the motivation instrument was app-referential and administered only once at posttest in the Treatment Group, motivation analyses were descriptive and were not compared between groups. Two-tailed p values are reported with α = 0.05. Effect sizes are reported as Cohen’s d for t tests and partial η² for mixed-design ANOVAs, together with 95% confidence intervals (95% CIs) for mean differences. Because multiple outcomes were tested, results close to the significance threshold are interpreted cautiously and emphasis is placed on effect sizes and the overall pattern of findings.

## Results

### Descriptive statistics and baseline comparison

#### Sample characteristics.

The demographic profile of the participants is presented in Table 3. The study included 90 first-year students, who were equally assigned to a treatment group (n = 45) and a control group (n = 45). The gender distribution was relatively balanced between groups, with females constituting the majority in both the Treatment Group (66.7%) and the Control Group (71.1%). Regarding age, the vast majority of participants (98.0%) were under 20 years old.

**Table 3:** Demographic information of samples.

Variable	Category	Treatment Group *(n = 45)*	Control Group *(n = 45)*
Gender	Male	15 (33.3%)	13 (28.9%)
	Female	30 (66.7%)	32 (71.1%)
Age	< 20	44 (98.0%)	44 (98.0%)
	20-25	1 (2.0%)	1 (2.0%)

#### Baseline physical fitness.

Prior to the intervention, independent-samples t tests were conducted to examine whether there were any baseline differences in physical fitness between the treatment and control groups (Table 4). No statistically significant differences were found for any of the four standardized fitness indicators. For example, pre-test scores on the 800/1000-meters run were 66.18 ± 10.48 in the treatment group and 64.27 ± 13.52 in the control group, t = 0.75, p = 0.456. Similarly, there were no significant between-group differences at baseline for 50-m sprint (t = 1.27, p = 0.209), standing long jump (t = 0.85, p = 0.398), or sit-and-reach flexibility (t = 0.31, p = 0.756). These results indicate that the two classes were comparable in terms of basic athletic ability at the outset of the experiment.

**Table 4:** Comparison of pre-test data between treatment and control groups.

Variable	Treatment Group (M ± SD)	Control Group (M ± SD)	t	p
50-meters	74.15 ± 9.70	71.64 ± 9.11	1.27	0.209
800/1000-meters	66.18 ± 10.48	64.26 ± 13.52	0.75	0.456
Sit-and-reach	74.78 ± 11.15	73.95 ± 13.72	0.31	0.756
Standing long jump	72.82 ± 12.20	70.56 ± 13.12	0.85	0.398

### Effects of the app-supported model on physical fitness

This section reports the statistical analyses for two distinct comparisons:

1. Within-Group Analysis: To verify if students made significant progress after the 10-week intervention.
2. Between-Group Analysis: To verify if the app-supported teaching model (Treatment Group) yielded superior final outcomes compared to the traditional model (Control Group).

#### Within-group changes in fitness scores.

Table 5 presents the results of paired-samples t tests comparing pre-test and post-test standardized fitness scores within each group. In both the control and treatment groups, 800/1000-meters endurance and standing long jump performance improved significantly over the 10-week period (control: t=−4.62, p < 0.001; t=−2.36, p = 0.023; treatment: t=−5.77, p < 0.001; t= −4.80, p < 0.001). In contrast, flexibility and sprint speed showed different patterns across groups. For sit-and-reach, no significant pre–post change was observed in the control group (t = 0.97, p = 0.337), whereas the treatment group exhibited a significant improvement (t=−2.60, p = 0.013). For 50-m sprint, the control group’s gains did not reach conventional significance (t= −1.93, p = 0.060), while the treatment group showed a significant improvement (t= −2.54, p = 0.015). Overall, these results indicate that both instructional models were associated with better endurance and explosive power, but the app-supported training model was additionally linked to significant gains in flexibility and sprint performance.

**Table 5:** Within-group comparison of pre-test and post-test scores.

Item	Group	Pre-test (M ± SD)	Post-test (M ± SD)	t	p
800/1000-meters	Control	64.27 ± 13.52	76.49 ± 10.50	−4.622	<0.001***
	Treatment	66.18 ± 10.48	78.96 ± 9.21	−5.772	<.0.001***
Standing long jump	Control	70.56 ± 13.12	76.91 ± 11.04	−2.360	0.023*
	Treatment	72.82 ± 12.20	82.89 ± 9.15	−4.800	<0.001***
Sit-and-reach	Control	73.96 ± 13.72	70.12 ± 24.27	0.971	0.337
	Treatment	74.78 ± 11.15	81.36 ± 12.53	−2.595	0.013*
50-meters	Control	71.64 ± 9.11	75.00 ± 6.78	−1.933	0.060
	Treatment	74.16 ± 9.70	79.16 ± 8.85	−2.537	0.015*

Note: *p < 0.05; **p < 0.01; ***p < 0.001.

#### Between-group differences in post-test fitness scores.

To evaluate the effectiveness of the intervention, independent-samples t tests were conducted on the posttest standardized fitness scores (Table 6). For endurance (800/1000-meters), there was no significant difference between the treatment group (M = 78.96, SD = 9.21) and the control group (M = 76.49, SD = 10.50), t = 1.19, p = 0.239, mean difference = 2.47, 95% CI [−1.67, 6.60], d = 0.25.

**Table 6:** Comparison of post-test scores between treatment and control groups.

Item	Treatment Group (n = 45, M ± SD)	Control group (n = 45, M ± SD)	Mean Diff.	95% CI for Diff	t	p	Cohen’s d
800/1000-meters	78.96 ± 9.21	76.49 ± 10.50	2.47	−1.67, 6.60	1.19	0.239	0.25
Standing long jump	82.89 ± 9.15	76.91 ± 11.04	5.98	1.73, 10.23	2.80	0.006**	0.59
Sit-and-reach	81.36 ± 12.53	70.12 ± 24.27	11.23	3.14, 19.33	2.76	0.007**	0.58
50-meters	79.16 ± 8.85	75.00 ± 6.78	4.16	0.85, 7.46	2.50	0.014*	0.53

Note: *p < 0.05; **p < 0.01; ***p < 0.001.

In contrast, the treatment group achieved higher posttest scores than the control group on standing long jump (M = 82.89 ± 9.15 vs. 76.91 ± 11.04, mean difference = 5.98, 95% CI [1.73, 10.23], t = 2.80, p = 0.006, d = 0.59), sit-and-reach flexibility (M = 81.36 ± 12.53 vs. 70.12 ± 24.27, mean difference = 11.23, 95% CI [3.14, 19.33], t = 2.76, p = 0.007, d = 0.58), and 50 m sprint performance (M = 79.16 ± 8.85 vs. 75.00 ± 6.78, mean difference = 4.16, 95% CI [0.85, 7.46], t = 2.50, p = 0.014, d = 0.53). These posttest comparisons suggest moderate between-group differences in favor of the app-supported training model for speed, explosive power, and flexibility, but not for endurance.

To account for the repeated-measures structure and evaluate whether the groups differed in change over time, we further conducted 2 (Group) × 2 (Time) mixed-design ANOVA for each outcome, focusing on the Group × Time interaction (Table 7). The interaction effect was significant only for sit-and-reach, indicating a clearer between-group difference in pre-to-post change for flexibility. For endurance, standing long jump, and 50 m sprint, the interaction effects were not significant, suggesting limited evidence that the posttest differences reflected differential improvement over time; thus, these outcomes should be interpreted primarily as between-group differences at posttest.

**Table 7:** Mixed-design ANOVA results for standardized physical fitness scores.

Outcome	df1	df2	F	p	Partial η²
800/1000-meters	1	88	0.03	0.872	0.000
Standing long jump	1	88	1.18	0.280	0.013
Sit-and-reach	1	88	4.93	0.029	0.053
50-meters	1	88	0.39	0.533	0.004

Because multiple posttest comparisons were conducted across four fitness indicators, a Holm–Bonferroni correction was applied. The adjustment did not change the overall pattern of results, and all substantive conclusions remained unchanged.

#### Exercise motivation in the experimental group.

Table 8 presents the descriptive statistics for intrinsic and extrinsic motivation. The results indicate high levels of motivation across both dimensions. The mean score for Extrinsic Motivation (M = 4.29) was slightly higher than Intrinsic Motivation (M = 4.22). Specifically, students reported the highest agreement with Item 6 (enthusiasm from progress, M = 4.51) and Item 3 (happiness from knowledge, M = 4.36). Each subscale means exceeded 4.0 on a 5-point scale, which is clearly above the scale midpoint (3.0), indicating generally high levels of both intrinsic and extrinsic motivation.

**Table 8:** Descriptive statistics for exercise motivation items (N = 45).

Dimension	Item Content	Mean	SD
Intrinsic Motivation	1.Using the app makes me more willing to exercise	4.11	0.885
	2. If time permits, I would use the app to learn more about sports	4.20	0.815
	3. Learning new knowledge in the app brings me happiness	4.36	0.830
	4. Through the app, I found new training methods/content	4.20	0.842
	5.Knowledge from the app inspires my confidence to participate	4.24	0.830
	Subscale Average	4.22	–
Extrinsic Motivation	6. When I make progress on the app, my enthusiasm increases	4.51	0.626
	7. The app encourages me to complete tasks within the given time	4.31	0.821
	8. My exercise motivation increases when using the app	4.29	0.815
	9. Even if I dislike PE, I can get good grades by following the app	4.11	0.959
	10. I want to get good grades to show my ability to others	4.22	1.042
	Subscale Average	4.29	–

Note: Items were rated on a 5-point Likert scale (1 = Strongly Disagree, 5 = Strongly Agree).

### Summary of hypothesis testing

H1: Students who participate in a Campus Sports World supported training model will show greater improvements in standardized physical fitness test scores than students who receive conventional college PE instruction without the app.

Result: Partially Supported. The Independent Samples t-test on post-intervention scores revealed mixed results across the four fitness indicators:

Supported: The Treatment Group achieved significantly higher post-test scores than the Control Group in explosive power (Standing long jump, p = 0.006), Flexibility (Sit-and-reach, p = 0.007), and Speed (50-meters sprint, p = 0.014).

Not Supported: For Endurance (800/1000-meter), the difference between the Treatment Group (M = 78.96) and the Control Group (M = 76.49) was not statistically significant (p = 0.239), although a positive trend was observed.

H2: Within the Treatment Group, mean levels of intrinsic and extrinsic motivation will be above the midpoint of the scale, reflecting student’ motivation profile in the app-supported condition.

Result: Observed. Descriptive analysis of the questionnaire data from the Treatment Group indicated that both Intrinsic Motivation (M = 4.22) and Extrinsic Motivation (M = 4.29) were above the midpoint of the five-point Likert scale and fell within the “Agree” range. This pattern suggests that students’ motivation in the app-supported condition reflected both interest in sports learning and achievement- or recognition-related considerations. A consolidated summary of the hypothesis testing outcomes is presented in Table 9.

**Table 9:** Final summary of hypothesis testing outcomes.

Hypothesis	Specific Indicators	Conclusion
H1: Physical Fitness	Speed (50-meters)	Supported (p < 0.05)
	Explosive power (Standing long jump)	Supported (p < 0.05)
	Flexibility (Sit-and-reach)	Supported (p < 0.05)
	Endurance (800/1000-meters)	Not Supported (p > 0.05)
H2: Motivation	Intrinsic Motivation	Supported (M > 4.0)
	Extrinsic Motivation	Supported (M > 4.0)

## Discussion

### The impact on basic athletic ability

Regarding the first research question, the app-supported model was associated with more favorable post-intervention performance on several standardized fitness outcomes. At posttest, the Treatment Group scored higher than the Control Group on the 50 m sprint, standing long jump, and sit-and-reach, whereas no significant difference was observed for the 800/1000 m run. When considering change over time, both groups showed significant within-group improvements in endurance and standing long jump, while only the Treatment Group showed significant gains in sprint speed and flexibility. Consistent with this pattern, the 2 (Group) × 2 (Time) mixed-design ANOVA indicated a significant Group × Time interaction only for sit-and-reach (Table 7), suggesting stronger evidence of differential improvement for flexibility than for the other outcomes. Therefore, the fitness findings should be interpreted as quasi-causal evidence in a class-level, non-randomized design, with particular caution for outcomes where the interaction effects did not reach significance.

From a task–technology fit (TTF) perspective [7], these results are plausible because the app provides structured task assignment, tutorials, and monitoring functions that map well onto skill-dependent requirements of speed, explosive power, and flexibility training. Video demonstrations, standardized technique cues, and timely completion feedback may reduce execution barriers during extracurricular practice and help students organize and refine their training, which is likely to benefit technique-sensitive tasks. By contrast, aerobic endurance adaptations depend more strongly on whether training intensity and volume reach an adequate physiological stimulus. In this study, Campus Sports World supported scheduling and volume logging but did not provide intensity prescriptions or objective monitoring (e.g., heart-rate zones or pace targets), and training intensity was likely self-selected. This implementation limitation, together with the relatively short 10-week timeframe, may explain why endurance did not differ between groups, and it cautions against generalizing the null endurance finding beyond the current dose and app features. Future work that incorporates progressive overload principles and objective intensity monitoring may be better positioned to target aerobic capacity, as emphasized in exercise prescription guidance and endurance training evidence [19–21].

### Exercise motivation in the app-supported condition

For the second research question, students in the Treatment Group reported high levels of both intrinsic and extrinsic motivation (Table 8). Because motivation was assessed only once at posttest and only among app users, these findings are descriptive and cannot be attributed to the intervention through between-group comparisons. Interpreted through self-determination theory (SDT) [9,10], the pattern is consistent with an app-supported setting that may be experienced as relatively need-supportive. At the same time, the instrument was assembled for an app-supported context and is intended to capture broad motivational orientations rather than fine-grained SDT regulations; some items (e.g., enthusiasm from progress or grade-related statements) may reflect identified or introjected reasons alongside extrinsic motives, so the intrinsic–extrinsic distinction should be interpreted with this overlap in mind.

Several app features offer a plausible context for this motivation profile. Progress visualization and performance feedback may make improvement more visible and strengthen competence; flexible completion windows can allow choice of when and where to train, supporting autonomy; and social functions such as class leaderboards and peer comparisons may increase relatedness by embedding individual effort in a shared class context. Given that app records contributed to course process evaluation, it is also plausible that achievement- and recognition-related considerations co-occurred with genuine interest in learning and exercising, yielding simultaneously high intrinsic and extrinsic motivation scores.

The observed motivation pattern can also be discussed in light of the trans-contextual model (TCM), which proposes that autonomous motivation supported in physical education can transfer to leisure-time physical activity [15]. In a sample of Chinese college students, perceived autonomy support in physical education has been associated with more autonomous motivation toward leisure-time physical activity [22], and related intervention work has shown that a TCM-based physical activity course can promote leisure-time physical activity behavior among university students [23]. Although the current study does not test motivational transfer pathways, the app-supported model extends PE into structured extracurricular practice and may provide a setting in which need-supportive experiences are carried into out-of-class exercise.

### Implications for practice

The findings have several practical implications for app-supported college PE. First, when the instructional goal emphasizes technique-sensitive components (e.g., sprint mechanics, jumping technique, and flexibility routines), apps that provide standardized tutorials, progress tracking, and timely feedback may help students complete extracurricular tasks more consistently and with clearer performance cues. Second, for endurance-oriented outcomes, distance logging alone may be insufficient; implementations that incorporate explicit intensity guidance (e.g., pace targets or heart-rate zones) and progressive overload planning, ideally supported by wearable-based monitoring, are more likely to deliver a training stimulus aligned with aerobic adaptation [19–21]. Third, when app records are used in summative or high-stakes evaluation, colleges should transparently communicate grading rules and ensure data governance and fairness, including clear policies on privacy, device access, and how abnormal records are handled.

Finally, motivation-oriented design should not rely solely on gamification or ranking. Combining an app-supported training model with need-supportive teaching practices delivered by PE teachers may provide a more robust approach to sustaining participation. For example, teachers can offer meaningful rationales, provide choice within tasks, use informational feedback, and avoid controlling communication—behaviors that have been synthesized into a practical classification system for SDT-informed interventions [24]. Embedding these practices alongside app use may help clarify how motivational experiences relate to adherence and longer-term fitness outcomes in future studies.

### Limitations and future research

This study has several limitations. First, the quasi-experimental design relied on two intact classes in a single institution, so selection bias due to unmeasured class differences cannot be ruled out, and generalizability may be limited by the specific cohort of first-year art and design majors. Second, the same instructor delivered both conditions; although this improves curricular comparability, it also introduces the possibility of instructor expectancy effects, and minor spillover between classes cannot be fully excluded. Third, motivation was assessed only once at posttest and only in the app-supported group because the questionnaire items explicitly referred to app use, which precluded pre–post assessment and between-group comparisons; thus, motivation results should be interpreted as a descriptive profile rather than intervention effects. Fourth, motivation relied on self-report. While the brief, app-tailored scale showed high internal consistency, its construct validity remains preliminary: the two-factor structure was supported only by exploratory factor analysis (see S1 Table), and several items may reflect overlapping SDT regulations (e.g., identified or introjected reasons) rather than purely intrinsic or extrinsic motives; further work should apply confirmatory factor analysis and related validation procedures in larger samples. Fifth, the intervention period was relatively short, and the app did not provide intensity prescription or objective monitoring (e.g., heart-rate zones or pace targets), so training load could not be quantified with objective indicators—an important constraint when interpreting the endurance findings. Finally, multiple outcomes were tested. Although mixed-design ANOVAs were conducted to evaluate Group × Time interactions and posttest comparisons were examined with Holm–Bonferroni adjustment, findings close to the significance threshold and outcomes with non-significant interaction effects should still be interpreted cautiously.

Future research should adopt randomized or cluster-randomized designs across multiple institutions, incorporate longer follow-up periods, and assess motivation repeatedly in both groups to enable stronger temporal and causal inferences. In addition to questionnaires, integrating behavioral indicators such as app usage logs and task completion records, together with objective training-load measures (e.g., wearable-derived heart rate, pace, or workload metrics), would help clarify whether observed fitness changes are driven by differences in adherence, training dose, or movement quality. From an analytical perspective, multilevel models (e.g., linear mixed models) are recommended to account for clustering at the class level and the repeated-measures structure, and preregistered analysis plans with prespecified primary outcomes and multiplicity control would further improve robustness. Measurement work is also warranted to strengthen the motivation instrument, including confirmatory factor analysis and tests of measurement invariance in larger and more diverse samples. Finally, future interventions could experimentally integrate SDT-informed, need-supportive teaching components alongside the app—such as providing meaningful rationales, offering choice within tasks, and using informational feedback—drawing on recent SDT intervention guidance that classifies teachers’ motivational behaviors into concrete, trainable strategies [24].

## Conclusion

This quasi-experimental study examined whether integrating the Campus Sports World app into a college PE course was associated with changes in students standardized physical fitness and with post-intervention motivation in the app-supported condition. Compared with conventional instruction, the Treatment Group showed higher posttest scores in speed, explosive power, and flexibility, while no posttest difference was observed for endurance. Mixed-design ANOVA indicated that the clearest evidence of differential pre-to-post change was for flexibility; group-by-time interactions for the other outcomes were not significant. These findings suggest that the current implementation may be better aligned with skill-related fitness components than with endurance development, which typically requires more explicit control of training intensity and volume.

Students in the Treatment Group reported high intrinsic and extrinsic motivation after the intervention. Because motivation was assessed only once and only in the app-supported group using app-referential items, these results are descriptive and cannot be attributed to the intervention. Future research should use multi-site randomized or cluster-randomized designs, include repeated motivation measures in both groups, incorporate objective indicators of training load and intensity, and extend follow-up to assess durability of effects.

## Supporting information

S1 Data Main study dataset. Anonymized participant-level data including group assignment, pre- and post-test physical fitness scores, and posttest exercise motivation responses used in the analyses.
(CSV)

S2 Data Exercise motivation questionnaire dataset. Item-level questionnaire responses and subscale scores for intrinsic and extrinsic motivation used in the manuscript.
(CSV)

S1 File Codebook for S1 Data and S2 Data. Variable names, labels, coding schemes, and scoring information for the datasets used in this study.
(XLSX)

S1 Table Exploratory factor analysis of the exercise motivation questionnaire (Treatment Group, N = 45). (DOCX)
