# Supplementary material for: App-supported versus conventional college physical education: Effects on standardized physical fitness scores and exercise motivation in Chinese university students
Source: PLoS One. 2026 Mar 23;21(3):e0345759. doi: 10.1371/journal.pone.0345759 (PMC13008050; doi:10.1371/journal.pone.0345759)
Supplement: S1 Table — (DOCX) [file pone.0345759.s004.docx]

# S1 Table. Exploratory factor analysis of the exercise motivation questionnaire (Treatment Group, N = 45)

| Item | Intrinsic Motivation | Extrinsic Motivation |
| --- | --- | --- |
| Using the app makes me more willing to exercise | 0.78 | 0.22 |
| I would use the app to learn more about sports if time permits | 0.74 | 0.25 |
| Learning new knowledge in the app brings me happiness | 0.81 | 0.19 |
| Through the app, I found new training methods/content | 0.76 | 0.27 |
| Knowledge from the app inspires my confidence to participate | 0.72 | 0.30 |
| When I make progress on the app, my enthusiasm increases | 0.28 | 0.71 |
| The app encourages me to complete tasks within the given time | 0.24 | 0.75 |
| My exercise motivation increases when using the app | 0.31 | 0.69 |
| Even if I dislike PE, I can get good grades by following the app | 0.18 | 0.77 |
| I want to get good grades to show my ability to others | 0.20 | 0.74 |

Note. Loadings ≥ 0.40 are considered salient.
